# Assessment of maxillary sinus lifting procedure in the presence of chronic sinusitis, a retrospective comparative study

**DOI:** 10.1016/j.amsu.2021.102379

**Published:** 2021-05-08

**Authors:** Abdullah Atef Hammuda, Mohamed Moawad Ghoneim

**Affiliations:** a Oral and Maxillofacial Surgery, Faculty of Dentistry, Suez University, Egypt; b Oral and Maxillofacial, Department of Oral and Maxillofacial Surgery, Faculty of Dentistry, Sinai University, Egypt

**Keywords:** Maxillary, Sinus lifting, Chronic sinusitis, Bone height, Infection, Healing scores

## Abstract

**Introduction:**

Chronic sinusitis can be considered a relative contraindication for sinus lifting procedure. However, its specific effects on bone height, infection and healing have not been as well investigated as its incidence as a post-operative complication.

**Methods:**

A retrospective comparative investigation was executed to evaluate the impact of chronic sinusitis on sinus lifting procedures regarding bone height, infection, and healing scores. Pre-operative and 6-month postoperative records (CBCT or CT and panoramic radiographs) of 40 patients who underwent sinus lifting procedure with graft and implant placement were split into two sets of 20 patients each; Group A is comprised of a patient with healthy sinus, whereas Group B includes patients with chronic sinusitis (identified as the presence of thickening of Schniederian membrane ≥ 2 mm, mucosal cyst, polyp or fluid level). Records of both groups were assessed for the difference in bone height of alveolar ridge between pre-operative radiograph and after six months postoperatively, and clinical notifications at the postoperative follow-up to report the healing and infection scores.

**Results:**

Statistically, there was non-significant difference in mean bone height gain (*p-value* > *0.05*) in comparison to control group mean bone height (8.84 ± 0.93). Also, there was non-significant variation in mean healing and infection scores.

**Conclusion:**

According to the available data, chronic sinusitis presenting as a thickening in the Schneiderian membrane has no significant effect on postoperative bone height, healing, or infection score in patients undergoing sinus augmentation with simultaneous implant placement. Further research is needed to better evaluate the effect of chronic sinusitis and its current status as a relative contraindication for sinus lifting procedure.

## Highlights

• Chronic sinusitis impact on sinus lifting in term of bone height, infection, and healing scores.
• Pre and postoperative assessment were conducted to evaluate this impact.
• Chronic sinusitis has no significant effect on postoperative bone height, infection or healing scores.
• The findings warrant further research into chronic sinusitis as a relative contraindication for sinus lifting procedures.

## 1 Introduction

Implant placement in the posterior maxilla is much more complicated than other jaws because of bone's quality and quantity. In alveolar bone deficiency, sinus lifting is usually used to insert an implant in the posterior maxilla [[1], [2], [3]]. The direct (lateral) and indirect (crestal) protocols was mentioned for sinus lifting because of residual alveolar bone height [[4], [5], [6], [7]].

Despite the method applied, post-surgical complications, e.g., graft failure, infections, and perforation of the sinus membrane, may eventually cause the surgical procedure's failure. Surgery's failure was thought to be linked with pre-existing sinus disease or sinus disease susceptibility [[8], [9], [10], [11], [12], [13], [14], [15], [16]]. Accordingly, pre-operative evaluation of the maxillary sinuses prior to augmentation is necessary to decrease post-operative complications.

Patients with chronic sinusitis can be considered as a relative contraindicated for sinus lift surgery and may have a possibility of acute postoperative sinusitis. That, modifying vulnerable physiology of chronic infected maxillary sinus damages the sensitive maxillary mucosal lining with surgical intention [17].

On the other hand, sinus lifting can be efficiently applied in asymptomatic sinus membrane pathology that does not cause obstruction or require surgical intervention [18].

A classification was proposed as a guideline for deciding when to perform an augmentation protocol, including sinus lift and graft technique. In this classification: category (1) including no radiographic pathology up to about 2 mm sinus lining thickening that is reliable with safe surgical augmentation. Category (2), with a 2–5 mm radiographic thickening, is not entirely contraindicated for sinus augmentation; it can be applied with caution, mainly if the thickening is in the high range. Category (3), with 6–9 mm radiographic thickening, is contraindicated for sinus augmentation with or without partial sinus obliteration. Category (4), with 6–9 mm radiographic thickening showing inflammation/infection by different causes, varying between odontogenic sinusitis to mucocele and is contraindicated for sinus augmentation [19].

In this study, A retrospective comparative investigation to appraise the impact of chronic sinusitis on sinus lifting procedures regarding bone height, infection, and healing scores.

## 2 Patients and methods

The current research was performed on 40 patients selected from oral and Maxillofacial outpatients’ clinics at the faculty of dentistry in Sinai, Suez Universities, and Egypt Maxillofacial center. All patients were subjected to open sinus lifting to increase alveolar ridge height for implant placement. The present study was planned as a comparative retrospective study under the World Medical Association Declaration of Helsinki, and was ethically permitted by research ethics committee of Faculty of Dentistry, Tanta University, Egypt, on February 2020***.*** The study was registered at Research Registry with the UIN: researchregistry6660 (registry#home/registrationdetails/604fca0c03dfdb001c21010d/) [20] and the work has been reported in line with the STROCSS criteria [21].

All clinical and radiographic data were acquired through the screening of saved patients' files and radiographs. The selected patients’ records fulfilled selective criteria including 1) Age ranging from 22 to 60 years, 2) Absence of systemic diseases or bone metabolism-related conditions, 3) Suitable oral hygiene, 4) Alveolar bone height ranged from 2 to 6 mm, 5) patients subjected to open sinus lifting procedures using B-tricalcium phosphate β-TCP grafting materials, 6) presence of pre-operative cone beam computerized tomography CBCT conventional CT, 7) the presence of sixth-month postoperative radiograph CBCT, CT or panoramic x-ray, 8) inclusion of postoperative follow up clinical data. Patients with 1) systemic diseases and/or conditions affecting bone or metabolism, 2) age above or below age limit 3) poor oral hygiene were excluded.

According to the inclusion criteria, 40 patients were selected and split into two groups: Group (A) comprised 20 patients with healthy maxillary sinus, and Group (B) had 20 patients with chronic sinusitis identified as the presence of thickening of Schniederian membrane ≥ 2 mm, mucosal cyst, polyp or fluid level.

## 3 Surgical procedures

All selected patients were subjected to surgical procedures performed under Local Anesthesia. The surgical procedures included lateral window sinus lifting procedure, augmentation using β-TCP, and implant placement.

All Surgical procedures were performed by Abdullah Hammuda using the same protocol.

- Topical betadine antiseptic solution was topically placed at site of LA injection and on the site of incisions, Local anesthetic nerve block (posterior superior alveolar nerve block and infraorbital nerve block) and local infiltration were injected in the posterior region of the maxilla using INIBSA ARTINIBSA Articaine 4% and adrenaline 1:100.000.®
- A crestal incision was performed on the alveolar ridge, slightly toward the palatal gingiva and then a full-thickness flap was raised by mucoperiosteal elevator to allow access to the lateral antral wall. Once the flap raised to the desired level, antrostomy performed with a round bur to create a trapdoor window on the lateral buttress of the maxilla at the site of sinus lifting. The sinus membrane then was gently lifted from the bony floor by a surgical curette, then lifting was performed on the lateral side and finally on the roof of the trapdoor.
- A space was created after the sinus membrane elevated, was grafted by a β-TCP mixed with antibiotic flumox® 1 g IV (500 mg amoxicillin sodium and 500 mg flucloxacillin sodium twice daily)
- postoperative medications protocol used was 1- (AUGMENTIN 1 g tablets: Each tablet contains 875 mg amoxicillin (as amoxicillin trihydrate) and 125 mg clavulanic acid (as potassium clavulanate), 2-(Flagyl ®:Metronidazole tablets 500 mg every 8 h, Sanofi), 3-(alphintern®: Chymotrypsin 300 E A U. (14 μkat, two tablets 3 times daily, Amoun) 4- (Celebrex®,celecoxib200 mg tablets once daily, Pfizer). 4-(Congestal® tablets once daily; Acetaminophen 650 mg, Chlorphenirarnine maleate 4 mg and Pseudoephedrine HCL 60 mg, Sigma).

## 4 Outcome measurement

Patients files and radiographs were analyzed to record the following.

- The difference in bone height of alveolar ridge between pre-operative radiograph and after six months postoperatively.
- Clinical notifications at the postoperative follow-up to record healing and infection scores [22,23].

## 5 Sample size and statistical analyses

To compare between groups (A) and (B), independent samples *t*-test or corresponding statistical analysis for nonparametric data is proposed. A total sample size of 40 samples will be sufficient to detect the effect size of 0.57, a power (1-β) of 80% (=0.80) at a significant level of p < 0.05. Group A and Group B will be represented by 20 patients. The sample size was calculated according to G*Power software version 3.1.9.5. [2, 3].

Reported data were investigated, analyzed by SPSS version 26.0 (SPSS Inc., Chicago, Illinois, USA). Data checked for normality using Shapiro-Wilk at 0.05 level. Accordingly, parametric data (The height bone gain, and infection scores) were presented as mean ± standard deviation (SD). Independent-t-test was applied to compare bone height, and infection scores, between two groups (control, and chronic sinusitis groups), and paired *t*-test for the differences between follow-up timepoints. Qualitative non-parametric data (healing scores) were presented as Median, IQ range, Mann-Whitney U were used to assess the difference between groups, and Friedman's test to evaluate differences between timepoints at a 0.05 significance level.

## 6 Results

**Fig. 1:**
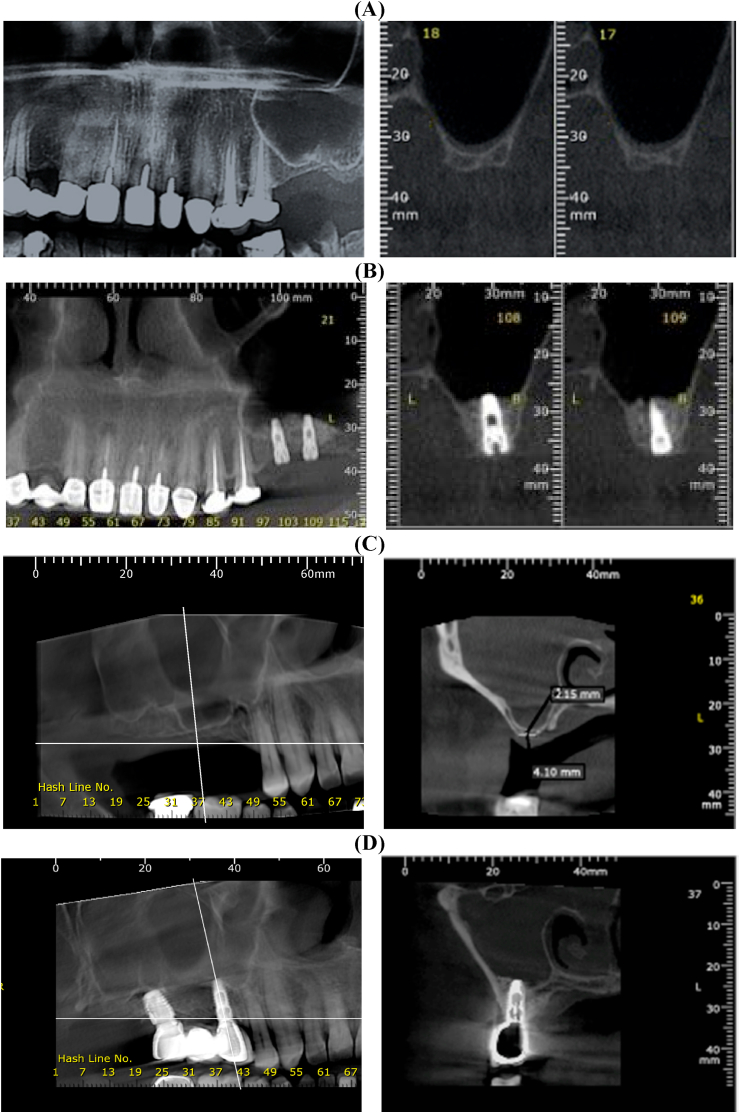
Cross-section on CBCT, (A) Preoperative showing normal sinus with low vertical bone height, (B) 6-month post-operative showing increase in bone height following osseointegrated implant and healing graft, and (C) pre-operative cross-section on CBCT showing chronic sinusitis with low vertical bone height. (D) 6-month post-operative cross-section on CBCT showing increased vertical bone height after osseointegrated implant and healing graft.

**Fig. 2:**
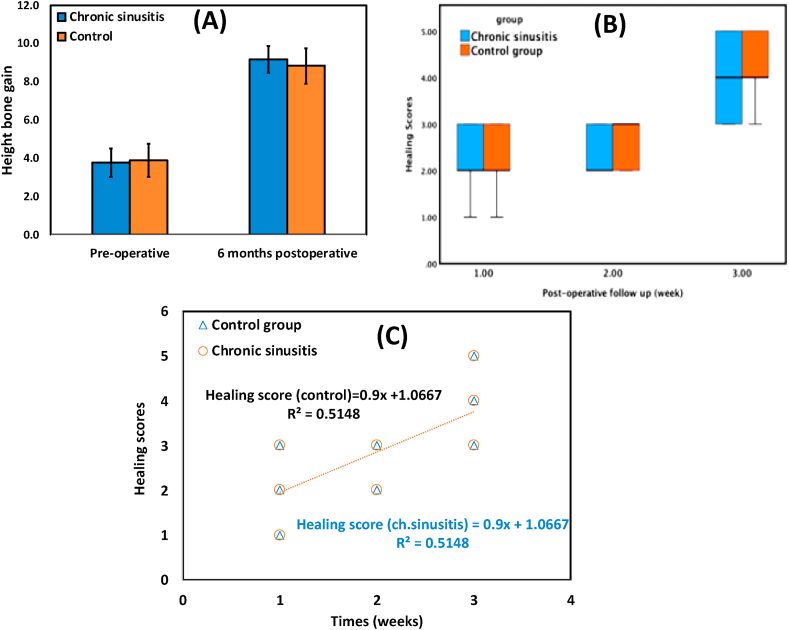
(A) bar chart of the height bone gain, (B) boxplot of the healing scores, and (C) regression trendline between postoperative follow-up time points (on X-axis) and healing scores (on Y-axis) in both chronic sinusitis and control groups.

Current research results are established and organized in the following tables and figures. The height bone gain, both pre-operative or postoperative (after six months), was presented in Table (1) and Fig. 1, Fig. 2. The height bone gain in the chronic sinusitis group showed an average (SD) of 3.78 ± 0.72 and 9.17 ± 0.69 in pre-and post-operative, respectively. However, In the Control group, the height bone gain showed an average (SD) of 3.89 ± 0.86 and 8.84 ± 0.93 in pre-and post-operative, respectively. The difference between the control and chronic sinusitis groups was statistically non-significant (p > 0.05), as revealed by the independent samples *t*-test. However, there was a statistically significant difference between pre-and post-operative (p < 0.05).

**Table 1:** Height bone gain in Chronic Sinusitis and Control Groups.

Height Bone Gain	Chronic Sinusitis Group *(n* = *10)*	Control Group *(n* = *10)*	*t*-test	p-value
**Preoperative**				
Mean ± SD	3.78 ± 0.72	3.89 ± 0.86	−0.31	0.760
Range	2.8–5	3–5.5		
**After 6months**				
Mean ± SD	9.17 ± 0.69	8.84 ± 0.93	0.90	0.378
Range	8.2–10.2	7.5–10.2		
**Diff. Pre and After 6m**				
Mean ± SD	5.39 ± 1.07	4.95 ± 1.10	0.91	0.376
Range	4–6.7	3.8–7		
Paired *t*-test	<0.05*	<0.05*		

Independent *t*-test; p-value>0.05 NS.

First week postoperative infection scores were recorded and presented in Table (2). The postoperative infection scores recorded and average of 7.70 ± 1.83 and 8.40 ± 1.35 in chronic sinusitis and control groups; respectively. Data shows non-significant difference between groups according to the first week postoperative infection score (t = *0.97; sign.>0.05).

**Table 2:** First-week postoperative infection scores in chronic sinusitis and control groups.

1st-week postoperative infection score	Chronic Sinusitis Group *(n* = *10)*	Control Group *(n* = *10)*	*t*-test	*p*-value
Mean ± SD	7.70 ± 1.83	8.40 ± 1.35	−0.974	0.343
Range	6–12	7–11		
Median	7.0	8.00		

t-Independent *t*-test; p-value>0.05 NS.

The healing scores in the first, second and third weeks postoperative according to healing score, was presented in Table (3) and Figure (2B, C). The healing score in the chronic sinusitis group showed an average (SD) of 2.20 ± 0.63, 2.40 ± 0.52, and 4.00 ± 0.82 in the first, second and third week operative; respectively. However, in the Control group, the healing score in the chronic sinusitis group showed an average (SD) of 2.20 ± 0.63, 2.70 ± 0.48, and 4.20 ± 0.63 in the first, second and third week operatively; respectively. The difference between the control and chronic sinusitis groups was statistically non-significant (p > 0.05). However, there was a statistically significant difference between follow up time points as revealed by Friedman's test for dependent samples(p < 0.05). Means followed by different letters are significantly different according to Bonferroni post hoc test at 0.05 level.

**Table 3:** Comparison between 1st-week Postoperative, second-week Postoperative, and third week Postoperative according to healing score in each group.

Post-operative timepoint (week)	Healing scores/Groups						Mann-Whitney U, Sign.
	Chronic Sinusitis Group			Control Group			
	Mean ± SD	Median	IQR	Mean ± SD	Median	IQR	
**1st**	**2.20** ± **0.63 b**	**2**	(2–3)	**2.20** ± **0.63 b**	**2**	(2–3)	>0.05 ns
**2nd**	**2.40** ± **0.52 b**	**2**	(2–3)	**2.70** ± **0.48 ab**	**3**	(2–3)	>0.05 ns
**3rd**	**4.00** ± **0.82 a**	**4**	(3–5)	**4.20** ± **0.63 a**	**4**	(4–5)	>0.05 ns
**Total**	**2.87** ± **1.04**	**3**	(2–4)	**3.03** ± **1.03**	**3**	(2–3.25)	>0.05 ns
**Friedman's test**	0.002**			<0.001***			

NS, non-significant at p > 0.05 NS; *, **. *** p-value<0.05, <0.010, <0.001.

## 7 Discussion

Several investigators have studied the prevalence of chronic sinusitis in patients scheduled for sinus augmentation, but with different results. Depending on radiographs (CT, and Panorama) and endoscopic examinations, Beaumont et al. reported that the presence of 40% sinus-related diagnoses in periodontal patients planned for sinus augmentation [24]. A prospective investigation of seventeen patients planned for sinus augmentation showed the existence of about 18% of patients with mucosal disease as revealed by pre-operative signs of sinusitis (clinical and radiographic), radiographic (Water's projection), and by endoscopic examination [25]. A study on 293 elderly patients (from 76 to 86 years old) claimed that 12% prevalence with sinus disease is revealed by panoramic radiographs [26]. There are various possible clarifications for the disparities among different investigations involving population features, pre-examination screening, inspection method, and standards defining sinus disease. Furthermore, sinus disease prevalence is more significant in the fall and winter seasons [27,28].

The thickness of normal sinus membrane has an average of 0.8 mm; it generally develops a thicker membrane with periodontal inflammation or chronic sinus or both. A sinus lining of more than 2 mm is considered relatively contraindicated; however, >5 mm is contraindicated [23] for sinus lifting [19,29].

In the current study, cases with membrane thickening ≥2 mm, mucosal cyst, polyp, or a fluid level were included in a chronic sinusitis group.

Various grafting materials was used in sinus augmentation protocol, including freeze-dried bone allografts, autogenous bone, alloplasts, xenografts, or a combination of them [2,30,31]. A recent study was carried on 119-patients revealed that a mean vertical bone gain of 8.5 ± 0.3 mm using β-TCP in maxillary sinus lift protocol by a lateral window method with instant implant placement for a follow-up of six-months [32].

In all selected cases, the present study in both groups was grafted with beta calcium triphosphate. The mean bone gain reported was 5.39 ± 1.07 and 4.95 ± 1.10 mm in both groups, respectively.

In Comparison between normal sinuses cases and chronic sinusitis cases, there were non-significant difference in mean bone height gain (*p-value* > *0.05*) compared with the control group mean bone height (8.84 ± 0.93). This observation revealed that, the presence of chronic sinusitis has no effect in sinus augmentation. It could be owing to the pre-existing thickening in the Schneiderian membrane may decrease the possibility of perforation and in absence of acute infection the healing of graft materials and osseointegration of implant would be not compromised.

These findings were coalescent with reported results of healing and infection scores, as in Comparison between both groups included in the current study revealed no statistically significant difference.

In agreement, a study evaluated maxillary sinus conditions prior to dental implantation by pre-operative sino-nasal evaluations. In a healthy population, it was stated that; the dental implant-related chronic rhinosinusitis risks is not high in patients with cysts, mucosal thickening or polyps of maxillary sinus. These patients could experience sinus augmentation and dental implant with close postoperative sinus follow-up.

In contrast another study reported that postoperative infection and implant loss occurred in 8/121 sinuses (6.6%) in correlation with pre-operative chronic sinusitis. The low failure rate (i.e 6.6%) may be referred to other factors related to patients medical status, implant stability, or grafting technique [33].

It was reported in many publications that, patients with pre-operative sinusitis significantly develop postoperative sinusitis. Nevertheless, between the incidental maxillary sinus findings on CT as mucosal thickening and a solitary cyst or polyp-like lesion are most frequent. The records of patients undergoing CT imaging for the maxillary sinus revealed that, 23.7%–28.2% mucosal thickening and 8.9%–19.4% cyst or solitary polyp and 3.6%–6.5% sinusitis [34].

Accordingly, many patients accidently discovered the maxillary sinus lesion can effectively obtain an implants or augmentation, and in several situations, their sinusitis recovers by medication and surgery. Though, its important to consider the relation between dental implant location and the maxillary sinus anatomy from sinus floor to the natural ostium to avoid ostium blockage by dental implants or grafts.

## 8 Conclusions

According to the available date chronic sinusitis presenting as thickening in the Schneiderian membrane has no significant effect on postoperative bone height, healing or infection score in patients undergoing sinus augmentation with simultaneous implant placement. It is recommended to perform further studies with large sample size to check these outcomes.

## 9 Limitations

The usual limitations inherent to retrospective studies are present in the current one. Attempts were made to alleviate some of them by reasonably widening the inclusion criteria, future prospective studies are needed to confirm the reached conclusions.

## Ethical approval

Ethics approval obtained by the Research Ethics Committee, Faculty of Dentistry, Tanta University, Egypt on February 2020.

## Sources of funding

Authors declare no involvement of study sponsors, or other sources of funding.

## Author contribution

All authors contributed equally in current research study.

## Registration of research studies

1. Name of the registry: https://www.researchregistry.com/browse-the-registry#home/
2. Unique Identifying number or registration ID: researchregistry6660
3. Hyperlink to your specific registration (must be publicly accessible and will be checked): https://www.researchregistry.com/browse-the-registry#home/registrationdetails/604fca0c03dfdb001c21010d/

## Guarantor

Abdullah Atef Hammuda.

Mohamed Moawad Ghoneim.

## Consent

Participants approval were collected prior to study. Written informed consent was obtained from the patient for publication of this case report and accompanying images. A copy of the written consent is available for review by the Editor-in-Chief of this journal on request”.

## Funding

The authors received no financial support for the research, authorship and/or publication of this article.

## Provenance and peer review

Not commissioned, externally peer-reviewed.

## Declaration of competing interest

No conflict of interest.
